# Association of hospital and health system factors with emergency department length of stay in older adults with dementia

**DOI:** 10.1186/s12873-025-01353-2

**Published:** 2025-09-26

**Authors:** Stephanie K. Nothelle, Eric P. Slade, Phillip D. Magidson, Laura Prichett, Amanda Finney, Tanya Chotrani, Halima Amjad, Sarah Szanton, Cynthia M. Boyd, Jennifer L. Wolff

**Affiliations:** 1 Division of Geriatric Medicine and Gerontology, Department of Medicine, Johns Hopkins University School of Medicine, Baltimore, MD USA; 2 Center for Aging and Health, Johns Hopkins University, Baltimore, MD USA; 3 Department of Health Policy and Management, Johns Hopkins Bloomberg School of Public Health, Baltimore, MD USA; 4 The Roger and Flo Lipitz Center to Advance Policy in Aging and Disability, Johns Hopkins Bloomberg School of Public Health, Baltimore, MD USA; 5 Johns Hopkins University School of Nursing, Baltimore, MD USA; 6 Department of Emergency Medicine, Johns Hopkins University School of Medicine, Baltimore, MD USA; 7 Biostatistics Epidemiology and Data Management Core, Johns Hopkins University School of Medicine, Baltimore, MD USA; 8 Division of Geriatric Medicine and Gerontology, Department of Health Policy and Management, Johns Hopkins University, 5200 Eastern Ave, MFL 7th Fl, Baltimore, MD 21224 USA

**Keywords:** Dementia, Emergency department, Length of stay, Boarding

## Abstract

**Background:**

Persons living with dementia (PLWD) have longer lengths of stay (LOS) in the Emergency Department (ED), which increases risk of delirium, falls and medication errors. Care of PLWD in the ED is complex and presence of dementia care specialists (geriatrics, neurology, psychiatry) may streamline care. We sought to understand the contribution of health system factors, including presence of dementia care specialists, to LOS among PLWD.

**Methods:**

We linked statewide ED visit data on patients discharged from the ED for Arkansas, Arizona, Florida and Massachusetts from the 2018 Healthcare Cost and Utilization Project State Emergency Department Database to the American Hospital Association Annual Survey and Healthcare Information Technology supplement. We included ED visit records for persons ≥ 65 years with ICD-10 dementia diagnoses. Median LOS was estimated at the hospital level and then used as a dependent measure in hospital-level Poisson multivariable models that conditioned on system characteristics.

**Results:**

We included 72,083 ED visits resulting in discharge at 225 health systems. Most EDs were in non-governmental, not-for-profit community hospitals (*n* = 159, 71%). Median patient age was 83 years (IQR 67, 92), females comprised a mean of 64% of visits. Median LOS was 4 h (IQR 3–7), mean LOS was 9.3 h (SD 16.3). Neurology was the most commonly available dementia care service (*n* = 180, 80%), followed by psychiatric services (*n* = 139 EDs, 62%) and geriatric services (*n* = 132, 59%). In Poisson models adjusting for a parsimonious set of co-variates, the presence of geriatric services was associated with a 16% lower mean LOS (IRR 0.84, 95% CI 0.73–0.97), however, this association lost significance in fully adjusted models (IRR 0.87, 95% CI 0.76–1.01).

**Conclusions:**

Availability of geriatric specialty services may offer hospitals an advantage in streamlining ED care for PLWD and in reducing visit length for this complex patient group. These findings reinforce the potential value of the Geriatrics Emergency Department Accreditation programs.

**Supplementary Information:**

The online version contains supplementary material available at 10.1186/s12873-025-01353-2.

## Introduction

Emergency department (ED) length of stay (LOS) is approximately 3 h longer for persons living with dementia (PLWD) than older adults without dementia [1]. While any visit to the ED can be fraught with risk, increased ED LOS increases the risk of medication errors, delirium, and death [2–5]. In fact, the new fiscal year 2025 Centers for Medicare and Medicaid Services inpatient prospective payment system includes protocols to reduce risk of ED delirium by reducing ED LOS and boarding as part of its new “Age Friendly Hospital Measures.” [6] Studies of general ED populations (i.e., not limited to PLWD or older adults) have identified a number of factors that can contribute to longer ED LOS [7–10]. Contextual factors such as hospital or health system-level factors are commonly identified as significant contributors to longer LOS [8, 11–13], however, the relationship between contextual factors and ED LOS has not been explored for PLWD.

The contextual factors that influence LOS for PLWD in the ED may be different than the contextual factors that influence ED LOS for the general population because of the unique challenges of caring for a PLWD in the ED [2]. Many clinicians report feeling unprepared to treat a PLWD [14, 15]. Availability of consultants who are trained in the care of PLWD may help streamline challenges and inefficiencies in care. Studies conducted within primary care have demonstrated that co-management of PLWD with a dementia-care specialist results in higher quality of care [16]. Studies of inpatient care have found that involvement of clinical nurses with advanced training in dementia care may reduce hospital LOS [17]. Whether involvement of specialists with training in dementia care within the ED could result in reduced LOS for PLWD is unknown.

The cognitive impairment that a PLWD experiences may limit their ability to accurately recall their medical history, recent symptoms or medications [14, 18]. Inability of the PLWD to report these important details about their care may heighten the importance health information technology and connectedness of electronic medical records across systems. For example, a recent primary care note for a PLWD who is presenting to the ED may reveal that the patient has dementia and that they were started on a medication that might explain their presenting symptoms. Such information could help an ED clinician more efficiently care for a PLWD during an ED visit. Use of health information from clinicians in other health systems or settings that do not share an electronic medical record (hereafter “outside health information”) in the care of PLWD has been associated with reduced risk of potentially preventable ED visits among PLWD and with shorter ED LOS in other populations, but the association between availability of outside health information and ED LOS among PLWD has not been explored [19–21]. 

We aim to examine the contribution of system level factors to ED LOS for PLWD. We specifically hypothesize that availability of specialists with expertise in dementia care and electronic availability of outside health information will be associated with significantly lower ED LOS for PLWD.

## Methods

### Data sources

We used three sources of data for this analysis. ED visit data was obtained from the 2018 Health Cost and Utilization Project (HCUP) State Emergency Department Database (SEDD) for Arizona, Arkansas, Massachusetts and Florida [22]. These states were chosen because they provided data on ED LOS in hours and minutes, allowing for more precise estimates of ED LOS. The SEDD captures information on all ED visits within a State that do not result in hospital admission [22]. Data elements reported vary across States, and include visit associated diagnoses and procedures, patient demographics (e.g. age, race, ethnicity, sex), payer, metro vs. non-metro location. The two other data sources were the 2018 AHA (American Hospital Association) Annual Survey and the AHA Healthcare IT (Information Technology) Survey [23, 24]. The AHA Annual Survey is a voluntary survey of all hospitals operating in the United States that collects information about a variety of hospital characteristics including organizational structure (e.g. for profit, government, not-for-profit), services provided, bed size, and staffing [24]. The AHA Health IT Supplement is a supplemental survey that is conducted annually by the AHA in collaboration with the Office of the National Coordinator and the Department of Health and Human Services. The survey collects information about electronic health record use, participation in and barriers to interoperability, and exchange of health information with external hospitals [23]. This study was determined to be exempt from full Institutional Review Board review in accordance with the Declaration of Helsinki, by the Johns Hopkins University School of Medicine IRB (0287523).

### Creation of study cohorts

**Fig. 1:**
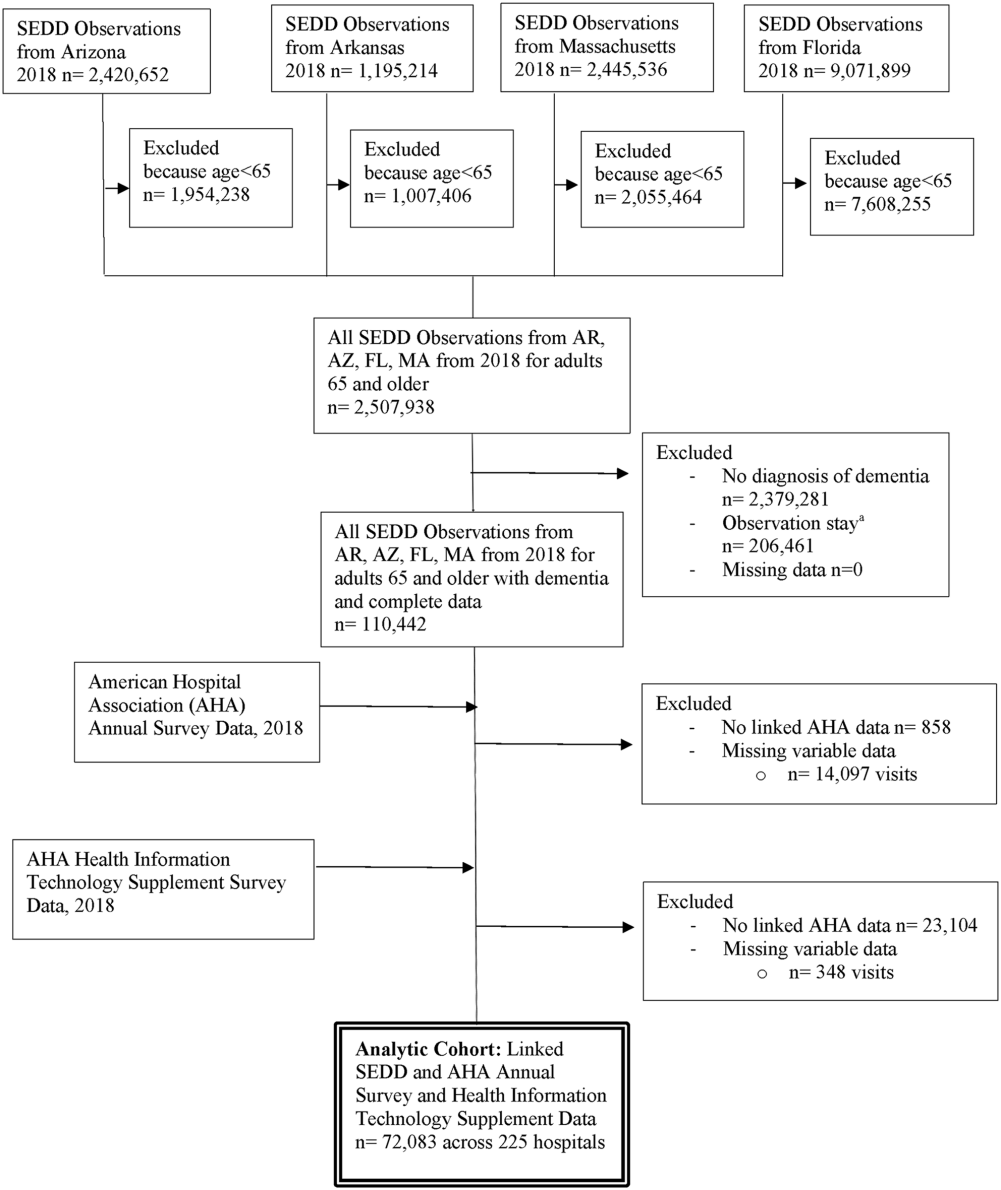
Flowchart of observation inclusion and exclusion into study sample

First, we pooled all ED visit observations from the 2018 SEDD for adults ages 65 and older from all four states (*n* = 2,507,938). (Fig. 1) We excluded ED visits by persons without dementia (*n* = 2,379,281) and visits that resulted in an observation stay (*n* = 206,461). We linked ED visit data from the SEDD to hospital characteristics in the AHA Annual Survey and AHA Healthcare IT Supplement using the AHA Hospital ID associated with each ED visit. We excluded ED visits which did not have linked AHA Survey Data (*n* = 858) or AHA Healthcare IT Supplement data (*n* = 23,104), or with missing data for the variables of interest (*n* = 348). This resulted in a sample of 72,083 ED visits within 225 EDs.

### Measures

Each measure was calculated at the level of the ED. We took this approach because the AHA survey provides characteristics of affiliated hospitals, meaning it was not possible to determine if a given service (e.g. geriatric care) was delivered at a given ED visit. For measures at the individual ED visit level, we pooled values for all visits within each ED and calculated the median (IQR) across EDs.

Our primary outcome measure was median ED LOS for an ED, which was reported in the SEDD in hours and minutes and converted to minutes for analysis. Visits by PLWD were identified by ICD-10 codes associated with each ED visit. As dementia is known to be under-coded in administrative data, we aimed to be broadly inclusive of dementia types; we combined lists of ICD codes from published sources (Table S1) [25–27]. 

We selected covariates based on the input-throughput-output conceptual model commonly applied to ED LOS [28], review of prior literature on ED LOS [7, 8, 11–13, 29, 30], and our prior study on ED LOS among PLWD [1]. Covariates from SEDD included age (median age at time of visit among those > 64 years and percent of visits by persons > 64), sex, race/Ethnicity, State the visit occurred in, whether the visit occurred during business hours (7a-6p), quarter of visit, primary payer (Medicare/Medicaid/Private Insurance/Other), Core Based Statistical Area (CBSA; metropolitan/non-metropolitan), discharge destination (community/transfer to skilled facility or another acute facility), clinical classification group for reason for visit (e.g. cardiac, infection) [31], number of Current Procedural Terminology (CPT) procedure codes and number of ICD-10 codes. Clinical classification group is a standardized taxonomy of ICD diagnosis codes into clinical categories for analysis [31]. We include race and Ethnicity to describe the diversity of the patient population represented and because of differences in access to care and ED LOS by race reported in prior studies [32–34]. 

Covariates from the AHA Annual Survey included: hospital ownership (federal government/government, non-federal/investor owned or for-profit/non-governmental and not-for-profit), ED visit volume, number of admissions, rate of admissions to the hospital from the ED, bed occupancy rate (the number of inpatient days divided by the number of hospital beds multiplied by 100), whether the hospital was a member of council on teaching hospitals and trauma center designation.

Presence of dementia care specialists refers to geriatric services, neurological services or psychiatric services (either psychiatric consultation or geriatric psychiatry services) in the hospital. Given that competence in caring for a patient population can also come from experience, we also calculated the volume of visits by PLWD within each ED [35]. 

Measures derived from the AHA Health IT Supplement include whether providers have necessary information from outside providers, and whether providers use patient health information received electronically from outside institutions.

### Analysis

The unit of analysis in this study was an ED. We used descriptive statistics to determine the distribution of covariates across included EDs. We then built individual multi-variable Poisson regression models for each of the six outcomes of interest- dementia care service (geriatric services, neurologic services, psychiatric services), volume of visits by PLWD, and health IT connectivity (whether providers have necessary outside information, and whether providers use electronically received outside information). Due to limited power based on our sample size (*n* = 225), we created two sets of parsimonious models with co-variates selected based on the input-throughput-output model, and prior studies on ED LOS [7, 8, 11–13, 28, 30, 33]. Model 1 adjusted for State of visit, volume of ED visits, number of CPT/HCPS procedure codes, metropolitan status, number of diagnosis codes, teaching hospital status, proportion of patients over 65, trauma center status. Model 2 additionally adjusted for race, top 5 reasons for visit, hospital ownership, volume of admissions and proportion of patients who were discharged to another facility (e.g. skilled nursing facility).

We conducted four sensitivity analyses. Poisson regression models were used to fit the sample distribution of LOS, which is right skewed. The sensitivity of the results to the Poisson distribution was explored by comparing the Poisson findings to results obtained from a generalized linear model with gamma distribution and log-link and a negative binomial model. During exploratory data analysis, we noted much higher LOS values in Arizona compared to other states, so we conducted a sensitivity analysis excluding Arizona. Second, since the HCUP SEDD indicator of observation status was not reliable, and thus our data could contain observation stays that were not identifiable, we conducted a sensitivity analysis excluding visits with a LOS > 90th percentile for the total sample [1]. All analyses were conducted in Stata 18.0 [36]. 

## Results

We included 72,083 ED visits not resulting in a hospital admission by PLWD over age 65 years at 225 hospitals in our analysis (Table 1). Median percentage of these ED visits by persons over 65 years at included EDs was 18% (IQR 14, 23). Median LOS was 240 min (IQR 180–420), while mean LOS was 557 min (SD 977). The top five reasons for visits by clinical classification group were injuries (e.g. sprain, fracture) 28% (IQR 22, 34), symptoms (e.g. syncope) 17% (IQR 14, 20), circulatory concerns (e.g. heart failure, hypertension) 7% (IQR 5,10), musculoskeletal concerns (e.g. arthritis, back pain) 5% (IQR 4,7), respiratory concerns (e.g. bronchitis, chronic obstructive pulmonary disease) 4% (IQR 3,5).

**Table 1:** Hospital-level characteristics among emergency department encounters with patients > 64 years and with a diagnosis of dementia, 2018

Characteristics		Total *n* = 225
State	Massachusetts, n (%)	35 (16%)
	Florida, n (%)	107 (48%)
	Arkansas, n (%)	50 (22%)
	Arizona, n (%)	33 (15%)
Age	Age of Visits in Cohort, median (IQR)	83 (82,84)
	Visits by persons over 65, median (IQR)	18% (14,23)
Sex	Proportion of Visits by females, median (IQR)	64% (60,67)
Race/Ethnicity	Proportion of Visits by Persons of White race, Median (IQR)	85% (67,92)
	Black, Median (IQR)	5% (2,13)
	Hispanic, Median (IQR)	4% (1,11)
	All others, Median (IQR)	1% (0,3)
Number of Diagnoses	Number of ICD-10 codes, Median (IQR)	7 (6,8)
Number of Procedures	Number of CPT/HCPS Codes, Median (IQR)	9 (8,11)
Primary Payor	Medicare, Median (IQR)	94% (91,96)
	Private insurance, Median (IQR)	2% (1,4)
	Other, Median (IQR)	2% (1,5)
Metropolitan Status^a^	Non-Metro, n (%)	43 (19%)
	Metro, n (%)	182 (81%)
Reason for Visit^b^	Injury, Median (IQR)	29% (22, 34)
	Symptoms not classified elsewhere, Median (IQR)	17% (14,20)
	Circulatory System Diseases, Median (IQR)	7% (5,10)
	Musculoskeletal System Diseases, Median (IQR)	5% (4,7)
	Respiratory System Diseases, Median (IQR)	4% (3,5)
	All others, Median (IQR)	36% (32,42)
Discharge Location	Routine discharge home/self-care, Median (IQR)	78% (67,84)
	Skilled Nursing Facility or other facility, Median (IQR)	22% (16,33)
Ownership	Government, non-federal, n (%)	23 (10%)
	Investor owned or for-profit	43 (19%)
	Non-governmental, not-for-profit	159 (71%)
Hospital Bed Occupancy Rate	Bed occupancy rate, Median (IQR)	60 (44,70)
Trauma Center	Certified trauma center, n (%)	130 (58%)
Number of admissions from ED	Admissions, Median (IQR)	9495 (3133,17193)
Rate of admissions from ED	Admission Rate (per 1000 ED visits), Median (IQR)	218 (142, 329)
Number of ED visits	Emergency department visit, Median (IQR)	35,559 (16368,61664)
Teaching hospital status	Member of Council of Teaching Hospitals, n (%)	23 (10%)
Volume of dementia patients^c^	Dementia, Median (IQR)	1 (1,1)
Dementia Care Services^d^	Geriatric Services, n (%)	132 (59%)
	Neurological Services, n (%)	180 (80%)
	Psychiatric Services including consultation/liaison, n (%)	139 (62%)
Health IT connectivity	Do providers routine have necessary information from outside providers electronically, n (%)	111 (50%)
	How frequently do providers use information received electronically from outside provide, n (%)	140 (63%)

ED = Emergency Department, IT = Information technology

^a^Based on the Core-Based Statistical Area ^b^Based on Clinical Classification Groupings. The top 5 most common Clinical Classification Groupings are presented, all others collapsed into “all others” ^c^Median number of visits with a dementia diagnosis in 2018 ^d^Services available in the hospital, health system or through a joint venture

Most of the included EDs (48%) were located in Florida and were non-governmental, not-for-profit facilities (*n* = 159, 71%). Neurologic services were the most commonly available dementia care services (*n* = 180, 80%), followed by psychiatric services (*n* = 139, 62%) and geriatric services (*n* = 132, 59%). 63% (*n* = 140) reported that providers used information received from outside providers often or sometimes, while 50% (*n* = 111) reported that providers routinely had the necessary information from outside providers electronically.

In Model 1, accounting for hospital state, number of ED visits, number of procedure codes, number of diagnostic codes, metropolitan location, bed occupancy rate, teaching status, volume of patients > 65 and trauma center status, measures of health IT connectivity including whether providers routinely had access to and used outside electronic information about patients were not associated with ED LOS, however presence of geriatric services was associated with approximately 16% shorter mean ED LOS (IRR 0.84, 95% CI 0.76–0.94). However, presence of neurologic services, psychiatric services and experience with dementia care were not associated with LOS. (Table 2, Table S2) In Model 2, when race, reason for visit, hospital ownership type, disposition of the patient and admission volume were additionally added to the model, the association between presence of geriatric services and LOS lost significance (IRR 0.87, 95% CI 0.76–1.01).

**Table 2:** Association between presence of dementia care services and health information technology connectivity with emergency department length of stay for persons with dementia, 2018

	Variables	Model 1^a^		Model 2^b^	
		IRR (95% CI)	*P*-value	IRR (95% CI)	*P*-value
Dementia Care Services^c^	Geriatric Services	0.84 (0.73, 0.97)	0.02	0.87 (0.76, 1.01)	0.06
	Neurological Services	0.97 (0.79, 1.19)	0.77	1.01 (0.82, 1.24)	0.96
	Psychiatric Services	1.08 (0.93, 1.25)	0.34	1.08 (0.96, 1.22)	0.21
Experience with dementia	Annual ED volume of visits by persons living with dementia	1.06 (0.92, 1.21)	0.43	1.09 (0.96, 1.24)	0.17
Health IT connectivity	Providers routinely have necessary information from outside providers electronically	1.10 (0.95, 1.27)	0.20	1.12 (0.97, 1.29)	0.12
	Providers frequently use information received electronically from outside providers	1.06 (0.90, 1.17)	0.69	1.06 (0.94, 1.20)	0.34

ED = Emergency Department, IT = information technology

^a^Model 1 adjusted for state, volume of ED visits, metro vs. non-metro status, bed occupancy rate, number of procedure codes, number of ICD codes, teaching hospital status, trauma center status, volume of patients > 64 years. ^b^Model 2 adjusted for Model 1 variables and race, reason for visit (Clinical Classification Group), discharge disposition, admission volume. ^c^Services available in the hospital, health system or through a joint venture

Sensitivity analyses using two alternative error distributions (negative binomial and gamma) produced similar coefficient values but had larger confidence intervals than the Poisson results presented in Table 2. (Table S3) Sensitivity analyses removing ED visits from Arizona found similar results to the main analysis with the additional finding of psychiatric services associated with approximately 20% longer mean LOS (Model 1 IRR 1.22, 95% CI 1.05–1.41; Model 2 IRR 1.21, 95% CI 1.04–1.42). (Table S4) Sensitivity analyses removing the top 10% of visits by LOS, to exclude observation stays, all prior associations lost significance but the direction of the associations remained unchanged. (Table S5)

## Discussion

In this four-state study of outpatient ED visits not resulting in hospital admission, the presence of geriatric services was associated with a 13% lower mean LOS for PLWD compared to EDs that did not have geriatric services available, though this finding was only significant in our parsimonious model. Other dementia care services and health IT connectivity were not consistently associated with LOS. In an era of increased ED crowding, and a growing aging population, understanding system-level factors associated with ED LOS can help target policies and interventions to improve care [37]. Our results suggest that health systems that operate geriatric services may have care processes that result in shorter LOS for PLWD, however, further research is needed to confirm this finding.

It is plausible that geriatrics services may result in a shorter LOS as previous studies have found that including clinicians with expertise in clinical care of older adults and PLWD may improve efficiency of care [38]. Comprehensive geriatric assessment has been associated with improved cancer care through improving communication about care planning and influencing oncologic treatment plans such that there is reduced toxicity of treatment [39, 40], and reduced LOS in the intensive care unit and hospital [17, 41]. Within the ED, among a general older adult population, geriatric specialists have been associated with reduced risk of admission and lower costs of care, but results regarding ED LOS have been mixed. Our study, is the first study, to our knowledge, on the involvement of geriatrics and ED LOS specifically for the population of PLWD [42–44]. Of note, we observed that although neurology and psychiatry are fields that also can provide expertise in dementia care, availability of these specialty services was not associated with shorter LOS. This may be due to the training of geriatricians in Internal Medicine or Family Medicine, meaning that geriatricians have the expertise and skills in a more holistic assessment of a PLWD in the ED, especially considering that most ED visits were not due to dementia related symptoms.

We observed that presence of geriatric services within a health system may be associated shorter ED LOS, though due to limitations of our data we cannot specify whether geriatrics involvement in any one visit is associated with ED LOS for that visit. Thus, if our observation that presence of geriatric services is associated with a shorter ED LOS for PLWD is true, it suggests that in addition to efficiency added by geriatric services in the care of individual older adult patients observed in other studies, presence of geriatric services in a health system may lead to spillover effects in workflow or processes that result in improvements in care. This finding emphasizes the importance of the ongoing work related to Geriatric Emergency Department Accreditation [45]. There are many models of Geriatric Emergency Departments, some with geriatrics trained specialists, others run by clinical support staff or non-geriatric trained physicians who have additional geriatrics expertise through non-fellowship training [46]. Evaluations of care at Geriatric Emergency Departments have been promising, showing improvements in costs and quality [42–44, 47–51]. Furthermore, these findings are particularly notable in light of the new Medicare Age Friendly Hospital measures which include a focus on reducing ED LOS to prevent adverse outcomes like delirium [6]. Our results suggest that inclusion of geriatric services may help achieve this metric.

We did not find a consistent significant association between ED LOS and health IT connectivity. In the more fully adjusted model, we did observe that having access to electronic information from outside providers was associated with an approximately 12% longer LOS. Others have found that the ability to access outside electronic information about patients in the ED leads to shorter LOS [20, 21, 52], however, these studies were not restricted to older adults or PLWD, who may have different care experiences. For PLWD, who have higher rates of health care use and multiple chronic conditions, increased access to information could help inform clinicians about the current state of care for a given PLWD but could also lead to information overload, which may compromise efficiency [53, 54]. Furthermore, access to outside electronic information may only be an issue in certain areas, such as urban centers with multiple competing health systems or in more rural areas with outpatient community sites that do not participate in information exchange. Our study is not able to determine whether external health information was available or used for an individual ED visit, but rather captures availability and use on an ED level. Given the implications of ED LOS for patient outcomes, care experiences and efficiency of care, more research is needed, including more contemporary research given the growth in interoperability and the higher rates of ED crowding in the post-COVID era [37]. 

The results of our sensitivity analyses also merit discussion. In our analysis that excluded data from Arizona, presence of geriatric services was no longer significantly associated with reduced ED LOS; however, presence of psychiatric services was significantly associated with a longer ED LOS. The loss of an association between geriatric services and LOS may have been due to a loss in power with fewer observations as the point estimate remained stable, while the confidence interval widened. The development of an association between psychiatric services and longer LOS was unexpected. We speculate this may be related to the known association of longer ED LOS for patients requiring psychiatric services, such that EDs that provide psychiatric services may disproportionately receive patients in need of psychiatric services, resulting in ED crowding and longer ED LOS for all ED patients. Hospital characteristics were not statistically significant in analyses that excluded the top 10% of LOS. This discrepancy suggests that our results were sensitive to the inclusion of people with prolonged ED LOS, who have a large influence on the average LOS.”

Our findings should be interpreted within the context of limitations. We only include data from four states, meaning that results may not be generalizable to all populations. However, we include Florida and Arizona which have a disproportionately high percentage of older adults and the included states represent different regions of the country. The percent of ED visits by persons of Black race was low, 5%, meaning results may not be generalizable to populations with a larger proportion of people of Black race. We identify dementia by ICD code, which may result in misclassification. Given the known under-coding of dementia we have likely miscategorized some PLWD with mild dementia as not having dementia and are likely capturing a population with moderate to severe dementia [55]. AHA data specify whether a service is available and the overall availability and use of outside electronic information, however, whether a given service was used for an ED visit is not clear.

In sum, we found that for PLWD who were discharged from the ED, presence of geriatric services within an ED’s health system may be associated with a shorter LOS, but that health IT was not consistently significantly associated with ED LOS among PLWD. Our results reinforce that care of PLWD in the ED may be influenced by different factors than that of other populations of patients visiting the ED, and that incorporation of geriatrics expertise may help improve efficiencies of care.

## Supplementary Information

Below is the link to the electronic supplementary material.

Supplementary Material 1

## Data Availability

This study used publicly available data from the Healthcare Cost and Utilization Project State Emergency Department Database (https://hcup-us.ahrq.gov/seddoverview.jsp) or for purchase from the American Hospital Association Annual Survey Database (https://www.ahadata.com/aha-annual-survey-database). Per data use policies from these organizations, the authors are not able to share the data.

## Abbreviations

ED: Emergency Department
LOS: Length of Stay
HCUP: Healthcare Cost and Utilization Project
CPT: Current Procedural Terminology
CBSA: Core Based Statistical Area
PLWD: Person living with dementia
IT: Information Technology
SEDD: State Emergency Department Database
AHA: American Hospital Association
