# Supplementary material for: Association of hospital and health system factors with emergency department length of stay in older adults with dementia
Source: BMC Emerg Med. 2025 Sep 26;25:193. doi: 10.1186/s12873-025-01353-2 (PMC12465944; doi:10.1186/s12873-025-01353-2)
Supplement: Supplementary file 1 — Supplementary Material 1 [file 12873_2025_1353_MOESM1_ESM.pdf]

Table S1. ICD-10 Diagnosis Codes for Dementia

ICD- 10 Code	Description
F0150	VASCULAR DEMENTIA WITHOUT BEHAVIORAL DISTURBANCE
F0151	VASCULAR DEMENTIA WITH BEHAVIORAL DISTURBANCE
F0280	DEMENTIA IN OTHER DISEASES CLASSIFIED ELSEWHERE WITHOUT BEHAVIORAL DISTURBANCE
F0281	DEMENTIA IN OTHER DISEASES CLASSIFIED ELSEWHERE WITH BEHAVIORAL DISTURBANCE
F0390	UNSPECIFIED DEMENTIA WITHOUT BEHAVIORAL DISTURBANCE
F0391	UNSPECIFIED DEMENTIA WITH BEHAVIORAL DISTURBANCE
F051	DELERIUM SUPERIMPOSED ON DEMENTIA
F1027	ALCOHOL DEPENDENCE WITH ALCOHOL-INDUCED PERSISTING DEMENTIA
F1097	ALCOHOL USE, UNSPECIFIED WITH ALCOHOL-INDUCED PERSISTING DEMENTIA
G300	ALZHEIMER'S DISEASE WITH EARLY ONSET
G301	ALZHEIMER'S DISEASE WITH LATE ONSET
G308	OTHER ALZHEIMER'S DISEASE
G309	ALZHEIMER'S DISEASE, UNSPECIFIED
G3101	PICK'S DISEASE
G3109	OTHER FRONTOTEMPORAL DEMENTIA
G311	SENILE DEGENERATION OF BRAIN, NOT ELSEWHERE CLASSIFIED
G3183	DEMENTIA WITH LEWY BODIES
A8100	CREUTZFELDT-JAKOB DISEASE UNSPECIFIED
A8101	VARIANT CREUTZFELDT-JAKOB DISEASE
A8109	OTHER CREUTZFELDT-JAKOB DISEASE
A811	SUBACUTE SCLEROSING PANENCEPHALITIS
A812	PROGRESSIVE MULTIFOCAL LEUKOENCEPHALOPATHY
A818	OTHER ATYPICAL VIRUS INFECTIONS OF CENTRAL NERVOUS SYSTEM
A8181	KURU
A8182	GERSTMANN-STRÄUSSLER-SCHEINKER SYNDROME
A8183	FATAL FAMILIAL INSOMNIA
G138	SYSTEMIC ATROPHY PRIMARILY AFFECTING CENTRAL NERVOUS SYSTEM IN OTHER DISEASES CLASSIFIED ELSEWHERE
G94	OTHER DISORDERS OF THE BRAIN IN DISEASES CLASSIFIED ELSEWHERE
F068	OTHER SPECIFIED MENTAL DISORDERS DUE TO KNOWN PHYSIOLOGICAL CONDITION
F05	DELIRIUM DUE TO KNOWN PHYSIOLOGICAL CONDITION
F061	CATATONIC DISORDER DUE TO KNOWN PHYSICAL CONDITION
G312	DEGENERATION OF THE NERVOUS SYSTEM DUE TO ALCOHOL
R54	AGE RELATED PHYSICAL DEBILITY
R4181	AGE RELATED COGNITIVE DECLINE
F04	AMNESTIC DISORDER DUE TO KNOWN PHYSIOLOGICAL CONDITION
G914	HYDROCEPHALUS IN DISEASES CLASSIFIED ELSEWHERE

Table S2. Complete Model Output for Model 1 Examining the Association between Dementia Care Services and Health Information Technology Connectivity and ED LOS

Panel A. Geriatric Services

		IRR	P-value	95% CI: Lower Bound	95% CI: Upper Bound
Geriatric Services		0.841	0.018	[0.728,	0.971]
Hospital State	MA	1.000			
	FL	1.013	0.905	[0.814,	1.262]
	AR	0.831	0.174	[0.637,	1.085]
	AZ	2.197	0.000	[1.726,	2.796]
Emergency room visit		1.000	0.000	[1.000,	1.000]
Number of CPT/HCPS Codes		1.073	0.000	[1.051,	1.096]
CBSA Type: Non-Metro		0.730	0.000	[0.612,	0.870]
Bed occupancy rate		1.000	0.310	[1.000,	1.001]
Number of ICD-10 codes		1.070	0.001	[1.026,	1.115]
Member of COTH		1.075	0.531	[0.857,	1.348]
Over 65		1.001	0.733	[0.993,	1.010]
Certified trauma center in hospital, health system and joint venture: Certified		0.991	0.888	[0.869,	1.130]
Intercept		2.005	0.006	[1.218,	3.298]

Panel B. Experience with Dementia Care

		IRR	P-value	95% CI: Lower Bound	95% CI: Upper Bound
Dementia		1.056	0.427	[0.923,	1.207]
Hospital State	MA	1.000			
	FL	1.002	0.986	[0.802,	1.252]
	AR	0.809	0.120	[0.619,	1.057]
	AZ	2.288	0.000	[1.812,	2.890]
Emergency room visit		1.000	0.001	[1.000,	1.000]
Number of CPT/HCPS Codes		1.075	0.000	[1.052,	1.099]
CBSA Type: Non-Metro		0.781	0.006	[0.655,	0.931]
Bed occupancy rate		1.000	0.064	[1.000,	1.001]
Number of ICD-10 codes		1.052	0.022	[1.007,	1.098]
Member of COTH		1.043	0.704	[0.839,	1.296]
Over 65		0.998	0.735	[0.985,	1.010]
Certified trauma center in hospital, health system and joint venture: Certified		0.963	0.604	[0.836,	1.109]
Intercept		2.086	0.008	[1.215,	3.581]

Panel C. Neurologic Services

		IRR	P-value	95% CI: Lower Bound	95% CI: Upper Bound
Neurological Services		0.969	0.768	[0.787,	1.194]
Hospital State	MA	1.000			
	FL	1.013	0.906	[0.813,	1.263]
	AR	0.814	0.133	[0.623,	1.064]
	AZ	2.235	0.000	[1.750,	2.853]
Emergency room visit		1.000	0.001	[1.000,	1.000]
Number of CPT/HCPS Codes		1.074	0.000	[1.050,	1.099]
CBSA Type: Non-Metro		0.765	0.005	[0.633,	0.924]
Bed occupancy rate		1.000	0.071	[1.000,	1.001]
Number of ICD-10 codes		1.062	0.005	[1.019,	1.108]
Member of COTH		1.027	0.805	[0.830,	1.270]
Over 65		1.001	0.873	[0.992,	1.010]
Certified trauma center in hospital, health system and joint venture: Certified		0.969	0.646	[0.845,	1.110]
Intercept		1.995	0.010	[1.178,	3.377]

Panel D. Providers have necessary information

		IRR	P-value	95% CI: Lower Bound	95% CI: Upper Bound
Providers have access to outside information		1.099	0.201	[0.951,	1.272]
Hospital State	MA	1.000			
	FL	1.016	0.885	[0.815,	1.268]
	AR	0.814	0.132	[0.622,	1.064]
	AZ	2.266	0.000	[1.776,	2.892]
Emergency room visit		1.000	0.001	[1.000,	1.000]
Number of CPT/HCPS Codes		1.074	0.000	[1.049,	1.099]
CBSA Type: Non-Metro		0.773	0.002	[0.655,	0.913]
Bed occupancy rate		1.000	0.052	[1.000,	1.001]
Number of ICD-10 codes		1.065	0.003	[1.022,	1.111]
Member of COTH		0.986	0.900	[0.794,	1.225]
Over 65		1.001	0.893	[0.992,	1.009]
Certified trauma center in hospital, health system and joint venture: Certified		0.999	0.988	[0.869,	1.149]
Intercept		1.805	0.027	[1.069,	3.047]

Panel E. Psychiatry Services

		IRR	P-value	95% CI: Lower Bound	95% CI: Upper Bound
Psychiatry Services		1.077	0.336	[0.926,	1.254]
Hospital State	MA	1.000			
	FL	1.031	0.785	[0.826,	1.287]
	AR	0.836	0.181	[0.642,	1.087]
	AZ	2.292	0.000	[1.806,	2.908]
Emergency room visit		1.000	0.001	[1.000,	1.000]
Number of CPT/HCPS Codes		1.073	0.000	[1.049,	1.098]
CBSA Type: Non-Metro		0.784	0.008	[0.657,	0.937]
Bed occupancy rate		1.000	0.044	[1.000,	1.001]
Number of ICD-10 codes		1.059	0.007	[1.016,	1.103]
Member of COTH		1.014	0.899	[0.817,	1.258]
Over 65		1.001	0.896	[0.992,	1.010]
Certified trauma center in hospital, health system and joint venture: Certified		0.952	0.520	[0.819,	1.106]
Intercept		1.915	0.012	[1.153,	3.180]

Panel F. Providers use outside information

		IRR	P-value	95% CI: Lower Bound	95% CI: Upper Bound
Providers use outside information		1.027	0.691	[0.901,	1.170]
Hospital State	MA	1.000			
	FL	1.050	0.665	[0.843,	1.306]
	AR	0.852	0.223	[0.658,	1.103]
	AZ	2.336	0.000	[1.844,	2.959]
Emergency room visit		1.000	0.000	[1.000,	1.000]
Number of CPT/HCPS Codes		1.076	0.000	[1.052,	1.100]
CBSA Type: Non-Metro		0.773	0.004	[0.648,	0.922]
Bed occupancy rate		1.000	0.075	[1.000,	1.001]
Number of ICD-10 codes		1.060	0.006	[1.017,	1.105]
Member of COTH		0.978	0.835	[0.790,	1.210]
Over 65		1.001	0.824	[0.992,	1.010]
Certified trauma center in hospital, health system and joint venture: Certified		0.966	0.591	[0.850,	1.097]
Intercept		1.846	0.020	[1.099,	3.099]

Table S3. Complete Model Output for Model 2 Examining the Association between Dementia Care Services and Health Information Technology Connectivity and ED LOS

Panel A. Geriatric Services

		IRR	P-value	95% CI: Lower Bound	95% CI: Upper Bound
Geriatric Services		0.871	0.061	[0.755,	1.006]
State	MA	1.000			
	FL	1.080	0.537	[0.845,	1.381]
	AR	0.854	0.198	[0.672,	1.086]
	AZ	2.154	0.000	[1.709,	2.715]
Emergency room visit		1.000	0.968	[1.000,	1.000]
Number of CPT/HCPS Codes		1.062	0.000	[1.039,	1.086]
CBSA Type: Non-Metro		0.750	0.004	[0.617,	0.911]
Bed occupancy rate		1.000	0.008	[1.000,	1.001]
Number of ICD-10 codes		1.071	0.001	[1.028,	1.115]
Member of COTH		1.050	0.713	[0.810,	1.360]
Over 65		1.002	0.573	[0.994,	1.011]
Certified trauma center		0.945	0.397	[0.828,	1.078]
Race: White		0.999	0.631	[0.996,	1.002]
Top 5 Reason for Visit		0.988	0.000	[0.981,	0.994]
	Government	1.000			
	For-profit	0.625	0.000	[0.495,	0.789]
	Not-for-profit	0.845	0.158	[0.668,	1.068]
Discharge home/self-care		1.002	0.619	[0.996,	1.008]
Admissions		1.000	0.235	[1.000,	1.000]
Intercept		5.092	0.000	[2.503,	10.359]

Panel B. Experience with Dementia Care

		IRR	P-value	95% CI: Lower Bound	95% CI: Upper Bound
Dementia		1.093	0.170	[0.963,	1.242]
State	MA	1.000			
	FL	1.097	0.461	[0.857,	1.404]
	AR	0.853	0.192	[0.671,	1.083]
	AZ	2.323	0.000	[1.856,	2.909]
Emergency room visit		1.000	0.769	[1.000,	1.000]
Number of CPT/HCPS Codes		1.068	0.000	[1.044,	1.091]
CBSA Type: Non-Metro		0.798	0.017	[0.663,	0.960]
Bed occupancy rate		1.001	0.001	[1.000,	1.001]
Number of ICD-10 codes		1.054	0.014	[1.011,	1.099]
Member of COTH		1.085	0.550	[0.830,	1.418]
Over 65		0.996	0.569	[0.984,	1.009]
Certified trauma center		0.915	0.203	[0.799,	1.049]
Race: White		1.001	0.698	[0.997,	1.004]
Top 5 Reason for Visit		0.986	0.000	[0.979,	0.992]
	Government	1.000			
	For-profit	0.592	0.000	[0.468,	0.750]
	Not-for-profit	0.807	0.073	[0.638,	1.020]
Discharge home/self-care		1.002	0.515	[0.996,	1.008]
Admissions		1.000	0.412	[1.000,	1.000]
Intercept		5.345	0.000	[2.668,	10.705]

Panel C. Neurologic Services

		IRR	P-value	95% CI: Lower Bound	95% CI: Upper Bound
Neurological Services		1.006	0.957	[0.817,	1.238]
State	MA	1.000			
	FL	1.096	0.481	[0.850,	1.412]
	AR	0.856	0.215	[0.669,	1.095]
	AZ	2.229	0.000	[1.754,	2.831]
Emergency room visit		1.000	0.848	[1.000,	1.000]
Number of CPT/HCPS Codes		1.064	0.000	[1.041,	1.088]
CBSA Type: Non-Metro		0.780	0.011	[0.643,	0.945]
Bed occupancy rate		1.001	0.001	[1.000,	1.001]
Number of ICD-10 codes		1.066	0.002	[1.023,	1.110]
Member of COTH		1.034	0.797	[0.799,	1.339]
Over 65		1.002	0.712	[0.993,	1.010]
Certified trauma center		0.921	0.221	[0.808,	1.051]
Race: White		1.000	0.982	[0.997,	1.003]
Top 5 Reason for Visit		0.987	0.000	[0.980,	0.994]
	Government	1.000			
	For-profit	0.594	0.000	[0.470,	0.750]
	Not-for-profit	0.808	0.071	[0.641,	1.018]
Discharge home/self-care		1.002	0.625	[0.995,	1.008]
Admissions		1.000	0.329	[1.000,	1.000]
Intercept		5.013	0.000	[2.470,	10.175]

Panel D. Providers have necessary information

		IRR	P-value	95% CI: Lower Bound	95% CI: Upper Bound
Providers have necessary outside information		1.119	0.117	[0.972,	1.287]
State	MA	1.000			
	FL	1.107	0.421	[0.864,	1.419]
	AR	0.843	0.176	[0.657,	1.080]
	AZ	2.247	0.000	[1.784,	2.832]
Emergency room visit		1.000	0.907	[1.000,	1.000]
Number of CPT/HCPS Codes		1.064	0.000	[1.040,	1.089]
CBSA Type: Non-Metro		0.779	0.005	[0.654,	0.929]
Bed occupancy rate		1.001	0.001	[1.000,	1.001]
Number of ICD-10 codes		1.072	0.001	[1.029,	1.116]
Member of COTH		0.993	0.959	[0.769,	1.283]
Over 65		1.001	0.812	[0.993,	1.009]
Certified trauma center		0.957	0.531	[0.835,	1.097]
Race: White		1.000	0.817	[0.997,	1.004]
Top 5 Reason for Visit		0.987	0.000	[0.980,	0.994]
	Government	1.000			
	For-profit	0.587	0.000	[0.465,	0.742]
	Not-for-profit	0.802	0.060	[0.638,	1.009]
Discharge home/self-care		1.001	0.737	[0.995,	1.007]
Admissions		1.000	0.317	[1.000,	1.000]
Intercept		4.627	0.000	[2.257,	9.487]

Panel E. Psychiatric Services

		IRR	P-value	95% CI: Lower Bound	95% CI: Upper Bound
Psychiatric services		1.084	0.314	[0.927,	1.267]
State	MA	1.000			
	FL	1.136	0.331	[0.879,	1.467]
	AR	0.881	0.307	[0.691,	1.124]
	AZ	2.308	0.000	[1.840,	2.895]
Emergency room visit		1.000	0.854	[1.000,	1.000]
Number of CPT/HCPS Codes		1.064	0.000	[1.041,	1.088]
CBSA Type: Non-Metro		0.799	0.014	[0.668,	0.955]
Bed occupancy rate		1.001	0.001	[1.000,	1.001]
Number of ICD-10 codes		1.064	0.002	[1.023,	1.106]
Member of COTH		1.035	0.792	[0.800,	1.339]
Over 65		1.001	0.843	[0.992,	1.010]
Certified trauma center		0.907	0.195	[0.781,	1.052]
Race: White		1.001	0.742	[0.997,	1.004]
Top 5 Reason for Visit		0.987	0.000	[0.980,	0.994]
	Government	1.000			
	For-profit	0.600	0.000	[0.476,	0.756]
	Not-for-profit	0.819	0.092	[0.649,	1.033]
Discharge home/self-care		1.002	0.586	[0.996,	1.008]
Admissions		1.000	0.355	[1.000,	1.000]
Intercept		4.605	0.000	[2.243,	9.453]

Panel F. Providers use outside information

		IRR	P-value	95% CI: Lower Bound	95% CI: Upper Bound
Providers use outside information		1.062	0.338	[0.939,	1.200]
State	MA	1.000			
	FL	1.130	0.344	[0.878,	1.455]
	AR	0.878	0.275	[0.694,	1.109]
	AZ	2.272	0.000	[1.806,	2.859]
Emergency room visit		1.000	0.997	[1.000,	1.000]
Number of CPT/HCPS Codes		1.064	0.000	[1.040,	1.088]
CBSA Type: Non-Metro		0.769	0.004	[0.645,	0.918]
Bed occupancy rate		1.001	0.000	[1.000,	1.001]
Number of ICD-10 codes		1.066	0.002	[1.024,	1.110]
Member of COTH		0.967	0.802	[0.746,	1.254]
Over 65		1.002	0.609	[0.994,	1.011]
Certified trauma center		0.926	0.234	[0.817,	1.051]
Race: White		1.000	0.839	[0.997,	1.003]
Top 5 Reason for Visit		0.987	0.000	[0.980,	0.994]
	Government	1.000			
	For-profit	0.578	0.000	[0.455,	0.734]
	Not-for-profit	0.786	0.044	[0.621,	0.994]
Discharge home/self-care		1.000	0.996	[0.994,	1.006]
Admissions		1.000	0.280	[1.000,	1.000]
Intercept		5.549	0.000	[2.773,	11.103]

Table S4. Results of generalized linear and negative binomial models for Association between presence of dementia care services and health information technology connectivity with Emergency Department length of stay for persons with dementia, 2018.

		Generalized Linear Model			Negative Binomial		
	**Variables**	Exp. Coefficient	P-Value	95% Confidence Interval	Exp. Coefficient	P-Value	95% Confidence interval
**Model 1**	Geriatric Services	0.89	0.10	[0.78, 1.02]	0.86	0.03	[0.74, 0.99]
	Neurological Services	1.04	0.68	[0.87, 1.23]	0.99	0.94	[0.81, 1.21]
	Psychiatric Services	1.13	0.08	[0.99, 1.28]	1.10	0.20	[0.95, 1.28]
	ED volume of dementia visits	1.08	0.20	[0.96, 1.22]	1.07	0.31	[0.94, 1.21]
	Providers routinely have necessary information from outside providers electronically	1.09	0.18	[0.96, 1.23]	1.10	0.18	[0.96, 1.26]
	Providers frequently use information received electronically from outside providers	1.04	0.51	[0.92, 1.18]	1.04	0.59	[0.91, 1.18]
**Model 2**	Geriatric Services	0.93	0.25	[0.82, 1.06]	0.88	0.09	[0.77, 1.02]
	Neurological Services	1.05	0.59	[0.88, 1.26]	1.02	0.87	[0.83, 1.25]
	Psychiatric Services	1.14	0.07	[0.99, 1.31]	1.11	0.21	[0.95, 1.29]
	ED volume of dementia visits	1.13	0.07	[0.99, 1.30]	1.10	0.14	[0.97, 1.26]
	Providers routinely have necessary information from outside providers electronically	1.13	0.05	[1.00, 1.28]	1.12	0.09	[0.98, 1.29]
	Providers frequently use information received electronically from outside providers	1.09	0.14	[0.97, 1.22]	1.07	0.26	[0.95, 1.21]

Exp= Exponentiated

Table S5. Association between presence of dementia care services and health information technology connectivity with Emergency Department length of stay for persons with dementia, in Florida, Massachusetts and Arkansas 2018.

		Model 1[a]		Model 2[b]	
	Variables	IRR (95% CI)	P-value	IRR (95% CI)	P-value
**Dementia Care Services[c]**	Geriatric Services	0.84 (0.73, 0.95)	0.007	0.87 (0.75, 0.99)	0.04
	Neurological Services	1.06 (0.87, 1.30)	0.56	1.03 (0.84, 1.26)	0.77
	Psychiatric Services	1.22 (1.05, 1.41)	0.01	1.21 (1.04, 1.22)	0.02
**Experience with dementia**	Annual ED volume of visits by persons living with dementia	1.12 (1.01, 1.25)	0.04	1.17 (1.05, 1.31)	0.01
**Health IT connectivity**	Providers routinely have necessary information from outside providers electronically	1.08 (0.95, 1.23)	0.23	1.18 (1.03, 1.36)	0.02
	Providers frequently use information received electronically from outside providers	1.07 (0.93, 1.22)	0.34	1.13 (0.98, 1.31)	0.08

ED= Emergency Department, IT= information technology

[a]Model 1 adjusted for state, volume of ED visits, metro vs. non-metro status, bed occupancy rate, number of procedure codes, number of ICD codes, teaching hospital status, trauma center status, volume of patients >64 years. [b]Model 2 adjusted for Model 1 variables and race, reason for visit (Clinical Classification Group), discharge disposition, admission volume. [c]Services available in the hospital, health system or through a joint venture

Table S6. Association between presence of dementia care services and health information technology connectivity with Emergency Department length of stay for persons with dementia, in Florida, Massachusetts, Arizona and Arkansas 2018 excluding those with the top 10% longest stays in each state.

		Model 1[a]		Model 2[b]	
	Variables	IRR (95% CI)	P-value	IRR (95% CI)	P-value
**Dementia Care Services**[c]	Geriatric Services	0.97 (0.85, 1.10)	0.63	0.95 (0.83, 1.10)	0.43
	Neurological Services	1.08 (0.90, 1.28)	0.41	1.07 (0.90, 1.3)	0.44
	Psychiatric Services	1.02 (0.89, 1.16)	0.80	0.99 (0.86, 1.14)	0.85
**Experience with dementia**	Annual ED volume of visits by persons living with dementia	1.03 (0.93, 1.14)	0.57	1.02 (0.92, 1.13)	0.74
**Health IT connectivity**	Providers routinely have necessary information from outside providers electronically	1.02 (0.90, 1.16)	0.80	1.03 (0.90, 1.17)	0.71
	Providers frequently use information received electronically from outside providers	1.06 (0.94, 1.20)	0.38	1.06 (0.94, 1.21)	0.35

ED= Emergency Department, IT= information technology

[a]Model 1 adjusted for state, volume of ED visits, metro vs. non-metro status, bed occupancy rate, number of procedure codes, number of ICD codes, teaching hospital status, trauma center status, volume of patients >64 years. [b]Model 2 adjusted for Model 1 variables and race, reason for visit (Clinical Classification Group), discharge disposition, admission volume. [c]Services available in the hospital, health system or through a joint venture
